# Optimizing iterative reconstruction for quantification of calcium hydroxyapatite with photon counting flat-detector computed tomography: a cardiac phantom study

**DOI:** 10.1117/1.JMI.8.5.052102

**Published:** 2021-03-10

**Authors:** Mikael A. K. Juntunen, Antti O. Kotiaho, Miika T. Nieminen, Satu I. Inkinen

**Affiliations:** a University of Oulu, Research Unit of Medical Imaging, Physics, and Technology, Oulu, Finland; b Oulu University Hospital, Department of Diagnostic Radiology, Oulu, Finland; c Medical Research Center, University of Oulu, Oulu University Hospital, Oulu, Finland

**Keywords:** computed tomography, coronary artery calcium, image quality, iterative reconstruction, phantom study, photon counting flat-detector

## Abstract

**Purpose:** Coronary artery calcium (CAC) scoring with computed tomography (CT) has been proposed as a screening tool for coronary artery disease, but concerns remain regarding the radiation dose of CT CAC scoring. Photon counting detectors and iterative reconstruction (IR) are promising approaches for patient dose reduction, yet the preservation of CAC scores with IR has been questioned. The purpose of this study was to investigate the applicability of IR for quantification of CAC using a photon counting flat-detector.

**Approach:** We imaged a cardiac rod phantom with calcium hydroxyapatite (CaHA) inserts with different noise levels using an experimental photon counting flat-detector CT setup to simulate the clinical CAC scoring protocol. We applied filtered back projection (FBP) and two IR algorithms with different regularization strengths. We compared the air kerma values, image quality parameters [noise magnitude, noise power spectrum, modulation transfer function (MTF), and contrast-to-noise ratio], and CaHA quantification accuracy between FBP and IR.

**Results:** IR regularization strength influenced CAC scores significantly (p<0.05). The CAC volumes and scores between FBP and IRs were the most similar when the IR regularization strength was chosen to match the MTF of the FBP reconstruction.

**Conclusion:** When the regularization strength is selected to produce comparable spatial resolution with FBP, IR can yield comparable CAC scores and volumes with FBP. Nonetheless, at the lowest radiation dose setting, FBP produced more accurate CAC volumes and scores compared to IR, and no improved CAC scoring accuracy at low dose was demonstrated with the utilized IR methods.

## 1 Introduction

In Agatston scoring, coronary artery calcium (CAC) content is quantified with unenhanced computed tomography (CT) to provide an overall coronary artery disease (CAD) grade.^1^ Agatston score is a well-established indicator of coronary artery plaque burden and the subsequent risk of cardiac events,^2^^,^^3^ and it has been proposed as a potential screening tool in asymptomatic patients.^2^^,^^4^ For a screening tool, though, the radiation dose of a CT protocol has to be effectively optimized, and consequently, several patient dose reduction approaches are being investigated. Recently, technical and computational improvements in hardware and algorithms have produced photon counting detectors (PCDs) and iterative reconstruction (IR) that could yield substantial reductions in radiation dose.^5^^,^^6^

The improved detection efficiency, reduced electronic and Swank noise, improved contrast, and higher spatial resolution with PCDs have motivated the development of prototype photon counting CT systems.^7^^,^^8^ Recently, a prototype PCD-CT system was utilized for CAC scoring.^9^ In that study, CAC scores agreed significantly better between standard dose clinical protocol and a low-dose protocol (75% dose reduction) using a PCD-CT system when compared to conventional EID-CT. Furthermore, with the same radiation dose, PCD-CT has allowed image noise reduction of 5% to 20% compared to current EID-CT technology.^7^^,^^10^^–^^15^

The emergence of IR has substantially influenced CT protocols. The vendors have their IR approaches for clinical CT, which in turn has resulted in substantial image quality improvements and radiation dose reductions in practice when compared to protocols utilizing the conventional filtered back projection (FBP) algorithm.^16^^,^^17^ These IR methods can operate in either projection or image domain, or they can yield the reconstruction through subsequent forward and backward projection operations (full statistical iterative or model-based iterative methods).^6^ Commonly, these full statistical IR algorithms minimize a cost function consisting of a data fidelity term, e.g., the $L_{2}$-norm of the residual, and a regularization term enforcing *a priori* information on the reconstruction task. Some frequently used full statistical IR methods in the inverse problems community utilize, e.g., total variation regularization^18^^,^^19^ or gamma regularization^20^ in the reconstruction. These methods are often computationally demanding, limiting their applicability in specific applications, such as trauma imaging, where reconstruction speed is vital. Nonetheless, IR approaches can effectively reduce noise magnitude and increase contrast-to-noise ratio (CNR), as has been demonstrated in the previous research.^21^^,^^22^ However, IR methods can produce distinct noise-texture, characterized by deviating noise power spectrum (NPS), compared to the traditional FBP.^23^ This deviating NPS can manifest as a “plastic” or “paint-brushed” appearance of reconstructions.^23^ For clinical applications, this phenomenon is often mitigated by blending FBP into the reconstruction.^24^

Apparent discrepancies exist in the literature regarding the performance of IR for CAC quantification. Several studies have reported significant differences between the calcium scores of IR and FBP reconstructions.^25^^–^^28^ In contrast, no significant differences between IR and FBP were observed in several analogous studies,^29^^,^^30^ although CAD risk reclassification was observed for a small subset.^29^ In another study, a risk reclassification rate of 13% with IR was deemed acceptable^31^ since it is generally between 10% and 11% with FBP when merely the scan starting position is changed.^32^^,^^33^ Because of these deviating results, CAC scoring is currently still performed with FBP. However, a comprehensive review concluded that modern IR algorithms could possibly perform low-dose CAC scoring reliably.^34^

Motivated by the reported potential for low-dose CAC scoring with PCD-CT,^9^ we investigate low-dose CT quantification of calcium hydroxyapatite (CaHA) with full statistical IR (hereafter referred to as IR) and a photon counting flat-detector CT (PC-FDCT) setup. Compared to the curved-array PCD, utilized with the aforementioned PCD-CT system, our flat-panel PCD has the same CdTe sensor material, bigger collimation (38.4 mm compared to 16 mm at the isocenter), and smaller pixel pitch of 100  μm in contrast to the 900-μm pixel size with PCD-CT system (4×4 matrix of 225-μm subpixels) (see Sec. 2.2. for further details).^9^^,^^35^^,^^36^ This combination of PC-FDCT with IR joins two effective dose reduction strategies and provides important benchmarking on the joint potential for patient dose reduction in CAC scoring. This assessment will be performed by imaging a cardiac rod phantom with an experimental PC-FDCT setup. With this simplified imaging model, we do not consider the motion of the heart, nor the attenuation in the torso, but rather focus on the impact of IR on CaHA quantification. Since we have addressed the utility of spectral PC-FDCT for quantification of CAC as previous research,^37^ in this study, we focus on low-dose CAC scoring. In contrast to the existing studies, we also compare the CAC scoring performance of IR at different regularization strengths to FBP using customized IR algorithms as they allow more flexibility in their regularization parameter selection than the proprietary algorithms found on commercial scanners. This comparison is performed at clinical and low-dose levels to address whether IR allows the reduction of dose without affecting the CAC scoring accuracy. To identify the sources of differences in CAC scoring performance between IR and FBP, a comprehensive analysis of technical image quality parameters [noise magnitude, noise texture, CNR, and modulation transfer function (MTF)] will be conducted. We hypothesize that suitable IR algorithms and optimally selected regularization strength will produce more accurate CAC scores compared to FBP at a low-dose protocol setting. Finally, as additional information for the readers, we will provide a comparison of image quality and radiation dose between clinical EID-CT scanner and our experimental PC-FDCT setup. However, since the assessment of radiation dose between PCD-CT and EID-CT has been assessed in several prior studies with more comparable and diagnostically relevant imaging setups,^7^^,^^10^^–^^15^ our comparison is provided as an appendix (Appendix B).

## 2 Materials and Methods

### 2.1 Imaging Phantom and Measurement Design

**Fig. 1:**
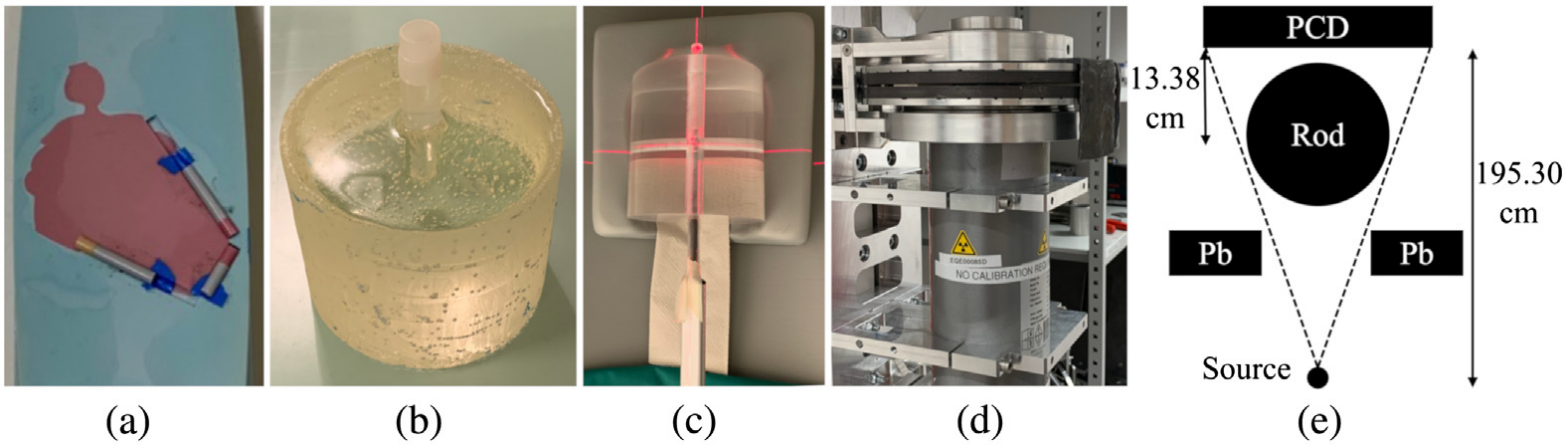
(a) Cross section of the cardiac rod phantom with CaHA inserts (red and yellow inserts) in the arteries; (b) epoxy resin phantom for noise texture optimization; and (c) dose measurement. The dose phantom was positioned using positioning lasers: (d) x-ray tube of the PC-FDCT setup and (e) schematic of the PC-FDCT setup. The x-ray tube allowed vertical collimation, whereas the horizontal collimation was obtained through lead (Pb) strips and blocks.

CaHA inserts (densities: 100, 250, and $400\;\mathrm{mg}/{\mathrm{cm}}^{3}$; diameters: 1.2, 3.0, and 5.0 mm; and a height of 7.0 mm) were placed in the coronary arteries of a cardiac rod phantom (008C, CIRS, Inc., Norfolk, USA) [Fig. 1(a)]. These inserts were selected as they covered the different CAD grades from no CAD to severe CAD (Table 5). Because of the small active area of the PCD ($5.13\times 15.47\;{\mathrm{cm}}^{2}$), we only imaged a cylindrical (9-cm diameter) cardiac rod and not the entire torso phantom with the experimental PC-FDCT setup [Fig. 1(e)]. A smaller rod phantom was chosen instead of the torso phantom to avoid interior tomography problem,^38^ which causes cupping artifacts in the reconstructed images^39^ that deteriorate the CaHA quantification accuracy. Also the rod was measured within a torso phantom with a clinical CT scanner to match the image noise texture between PC-FDCT and clinical CT CAC scoring protocol (see Sec. 2.3 for further details).

### 2.2 Experimental Photon Counting Flat-Detector CT Setup

The experimental PC-FDCT setup was constructed by mounting a PCD (XC-Flite FX15, XCounter AB, Danderyd, Sweden) on a table and fixing the center of rotation of a motorized rotation stage (NR360S/M, Thorlabs, Inc., Newton, New Jersey, USA) to the center of the active area of the PCD with a laser designator (Fig. 1). The x-ray tube (MXRP-160C, COMET Group, Flamatt, Switzerland) with a focal spot of 0.4  mm×4.0  mm (height×width) and inherent filtration of 0.5  mm Ti+2.0  mm $H_{2}0+2.0\;\mathrm{mm}$ Al was positioned 195.30 cm from the detector [Figs. 1(d) and 1(e)]. We set the isocenter-to-detector distance to 13.38 cm. The pixel pitch of the flat-panel PCD was 100  μm, and it covered a total active area of 5.13  cm×15.47  cm (height×width). The detector panel was an assembly of 24 separate tiles, each with 256×128  pixel area. We set the energy threshold of the PCD at 10 keV to remove electronic noise and used charge sharing correction. The frame rate of the PCD was 60 Hz, and the angular velocity of the motorized rotation stage was set to 4  deg/s resulting in 15 frames per angular degree. Therefore, we obtained reconstructions at 15 different dose levels from the PC-FDCT measurements by averaging different numbers of frames per degree. These different numbers of frames per degree, or simply frame averages, yield measurements at different doses and FBP noise levels. For each of these dose levels, we had 360 projections with one angular degree increments, which were subsequently reconstructed (see Sec. 2.4).

Especially with tiled flat-panel PCDs, the slightly deviating energy responses between tiles and pulse pile-up effects can lead to count-variations throughout the detector that will greatly deteriorate the image quality.^37^^,^^40^ Since PCDs require pixel-wise calibration of the panel beyond the simple flat-field correction,^40^^,^^41^ we applied the signal-to-equivalent thickness calibration (STC) method. In STC, increasing calibration material thicknesses are measured to obtain a correction map for measured counts.^41^^,^^42^ We used polymethyl methacrylate (PMMA) plates with thicknesses (5.35, 7.35, 10.75, and 12.75 cm) because of the similar mass attenuation and x-ray scattering properties between PMMA and soft tissues.

### 2.3 Imaging Protocols for CaHA Quantification

**Fig. 2:**
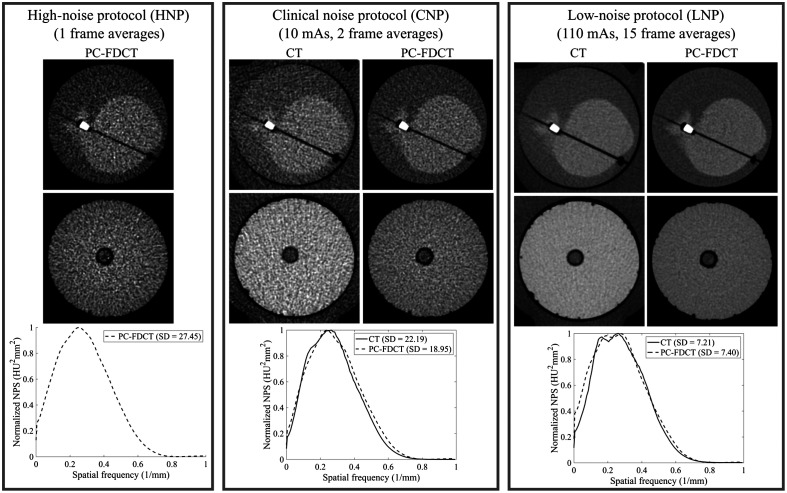
CT and PC-FDCT reconstructions of the cardiac rod and epoxy resin phantoms using FBP with similar image noises (HNP, CNP, and LNP) and their corresponding normalized NPSs with noise SD. With CT, the entire torso phantom is measured, but with PC-FDCT, only the rod phantom was imaged. Calcification with a density of $400\;\mathrm{mg}/{\mathrm{cm}}^{3}$ and a 5.0-mm diameter is visualized, and the central slice is visualized for the epoxy resin rod. Windowing was set at [−100,150]  HU. Since the scanner did not allow lower exposure than 10 mAs, we could not obtain the clinical HNP reference, and consequently, only the PC-FDCT reconstruction is visualized for HNP. The dark border surrounding the rod phantoms in the CT data originates from a small air gap between the rod and the torso phantom.

To assess the possible radiation dose reductions in CAC scoring with IR, we evaluated the CaHA insert volumes and scores with three different FBP noise levels. For a detailed description on the evaluation of image noise, see Sec. 2.6. The clinical noise protocol (CNP) with PC-FDCT was selected to produce comparable noise magnitude with FBP (${\mathrm{SD}}_{\mathrm{CNP}}=19.0\;\mathrm{HU}$) to the guideline CAC scoring noise level of 20 HU.^43^ Our high-noise protocol (HNP) (${\mathrm{SD}}_{\mathrm{HNP}}=27.5\;\mathrm{HU}$ with FBP) was selected to be comparable to that of a previous IR CAC scoring study (${\mathrm{SD}}_{\mathrm{HNP}}=27.3\;\mathrm{HU}$).^30^ Finally, we used a low-noise protocol (LNP) with a noise magnitude of 7.4 HU with FBP to provide reference values for CaHA insert volume and CAC score (Fig. 2).

In order to find a suitable FBP kernel with comparable noise texture between PC-FDCT and the clinical CAC scoring protocol, we used clinical CT (Somatom Definition Flash, Siemens Healthcare, Erlangen, Germany) as a reference and optimized the FBP reconstruction kernel for PC-FDCT (see Sec. 2.4 for further details). Clinical CT measurements were performed in axial mode to make the measurement geometry comparable with PC-FDCT. We measured a torso phantom (008C, CIRS, Inc., Norfolk, USA) with a clinical acquisition using two different noise levels: (1) CNP (exposure=10  mAs, CTDIvol=0.67  mGy) and (2) LNP (exposure=110  mAs, CTDIvol=7.42  mGy) (Fig. 2). Since the scanner did not allow lower exposure than 10 mAs, we could not obtain the clinical HNP reference. In the clinical Agatston scoring protocol, the manufacturer’s B35f convolution kernel was used (Table 1).

**Table 1:** Imaging parameters with clinical CT (clinical CAC scoring protocol) and PC-FDCT. Demonstration heartbeat setting was utilized when selecting the CT protocol parameters.

System	kVp	Exposure (mAs)	Scan time (s)	Filtration	Source-to-detector distance (cm)	Object-to-detector distance (cm)	Voxel size	Reconstruction method + kernel	Slice thickness (mm)	Collimation (cm)
CT^a^	120	10, 110	0.50	0.3 mm Ti + 1.0 mm C + 0.5 mm Al	108.56	49.06	0.32 mm×0.32 mm×1.5 mm	FBP + B35f	3.0	3.84
PC-FDCT	120	12.3, 24.6, 184.5	90	$0.5\;\mathrm{mm}\mathrm{Ti}+2.0\;\mathrm{mm}H_{2}0+2.0\;\mathrm{mm}$ Al	195.30	13.38	0.32 mm×0.32 mm×1.5 mm	FBP + moving average and Hann filter	3.0	3.84±0.1^b^
								IR		

a The imaging parameters were taken from a clinical CAC scoring protocol, and the helical scan mode was changed to axial scanning.

b The collimation for PC-FDCT is reported as mean ± error since we estimated the collimation from the measured projection data.

Agatston score, which is a product of density factor and calcification area (Table 2), is calculated for each CT slice separately, and the total calcium score is the sum of slice-specific scores.^1^^,^^44^ We segmented the CaHA inserts from the reconstructions using a fixed threshold of 130 HU and calculated the Agatston score for each slice according to Table 2. We obtained the CAC volume, and Agatston score reference values from the FBP reconstructed LNP measurements.

**Table 2:** Agatston score for CAD grading.

Maximum HU	Density factor	Total calcium score	CAD grade
<130	0	0	No CAD
130 to 199	1	1 to 10	Minimal
200 to 299	2	11 to 100	Mild
300 to 399	3	101 to 400	Moderate
≥400	4	>400	Severe

### 2.4 Image Reconstruction with PC-FDCT

For PC-FDCT reconstruction, we used the FBP algorithm from the ASTRA-toolbox (v. 1.8, iMinds-Vision Lab, University of Antwerp, Belgium) in MATLAB (v. 9.5, The MathWorks Inc., Natick, MA, R2018b) and optimized a custom FBP filter to produce similar noise texture to the clinical B35f kernel. We optimized the texture by matching the NPSs obtained from the CT and PC-FDCT LNP reconstructions of a homogeneous epoxy resin phantom (Fig. 2). The resulting FBP kernel for PC-FDCT was a combination of Hann-filter and moving average filter with a window length of 3.8 pixels (Table 1). This kernel produced the most comparable NPS with that of the clinical CAC scoring protocol.

The IR methods in this study were based on the generic regularized IR problem formulation:

$$\underset{u}{\arg\;\min}\;\frac{{\left\|Au-f\right\|}^{2}}{2}+\lambda R(u),$$ (1)

where the data-fidelity term minimizes the $L_{2}$-norm between the measured sinogram (f) and the forward model Au. A is the forward operator (projection matrix) defining the measurement geometry and u is the reconstruction. The regularization functional (R(u)) introduces *a priori* knowledge to the reconstruction task, whereas the regularization parameter (λ) balances the weighting between data-fidelity and regularization. The ASTRA and SPOT (v. 1.2) toolboxes^45^^,^^46^ were used to generate the projection matrix (A). Each slice was reconstructed separately in 2D with the fan-beam geometry.

In this study, we compared two commonly used regularization functionals: total variation (TV) and gamma (GAMMA) regularization. We chose these methods because of their prevalence in prior research.^18^^,^^19^^,^^47^ In summary, TV enforces piecewise smoothness through $L_{1}$-regularization of the gradient of the reconstruction.^19^^,^^48^ The smooth TV functional over the reconstruction domain (Ω) is formulated as

$$\mathrm{TV}(u)=\int_{\Omega }{\left({\left\|\nabla u\right\|}^{2}+\varepsilon \right)}^{1/2}dx,$$ (2)

where ε is a smoothing parameter ($10^{-8}$), ∇ is the gradient operator, and x is the coordinate vector. We discretized the TV-regularized minimization problem^19^ and obtained the converged solution using the gradient-descent method with Barzilai–Borwein step-size update^49^ with 500 iterations.

We formulated the gamma regularization functional as

$$\Psi_{\Gamma }(u)=\overset{N_{1}}{\underset{i=1}{\sum }}\overset{N_{2}}{\underset{j=1}{\sum }}\int_{0}^{{\left({\left|\nabla_{v}u_{i,j}\right|}^{2}+{\left|\nabla_{h}u_{i,j}\right|}^{2}\right)}^{1/2}}\frac{x^{\alpha -1}\beta^{\alpha }e^{-\beta x}}{\Gamma (\alpha )}dx,$$ (3)

where the shape and scale parameters α and β balance $l_{1}$- and $l_{0}$-norms in regularization, $N_{1}$ and $N_{2}$ are the reconstruction dimensions with $\nabla_{v}$ and $\nabla_{h}$ denoting their respective directional gradients, and Γ denotes the gamma function.^20^ Gradient descent method with 500 iterations was used to ensure convergence in the gamma-regularized reconstructions. The number of iterations for TV and gamma regularization was selected using a convergence check (see Appendix A for further details). All IR image data in this work are from 500 iterations. The IR methods were initialized with a zero matrix.

### 2.5 Regularization Parameter Selection

The regularization parameter directly affects the reconstruction noise magnitude, noise texture, and image sharpness. As it influences the reconstruction quality and CAC scores, we compared two different regularization parameter selection approaches in this study as follows.

(1) *Fixing the noise magnitude for reconstructions obtained from different frame averages*. With this approach, regularization is increased at lower radiation doses to preserve the noise magnitude. We fixed the noise magnitude for each frame average to that of the LNP, i.e., 7.4 HU (see Sec. 2.3). The stability of noise level with respect to different frame averages with adaptive IR regularization strength was determined by calculating a set of IRs with different regularization parameters for one PC-FDCT slice as prior work. From these reconstructions, we selected the regularization strength that yielded the closest noise level to the preselected level of 7.4 HU.
(2) *Fixing the regularization parameter to the same value for each dose level within a reconstruction method*. Therefore, the extent of regularization is consistent for different frame averages, but the noise magnitude will increase with reducing number of averaged frames. Three different fixed regularization strengths were selected (denoted as strong, medium, and weak regularization). The medium regularization strength was selected to visually match the MTFs between IR and FBP reconstructions for the LNP. Having three different fixed regularization strengths assesses how regularization strength affects CAC volumes and scores.

The regularization parameters for the IR algorithms are summarized in Table 3. For simplicity, we fixed the shape and scale parameters for gamma regularization (α=1.2 and β=0.6) and only modified the regularization strength [λ in Eq. (1)].

**Table 3:** Selected regularization parameters for different frame averages (noise levels). The reference noise levels for the protocols (LNP, CNP, and HNP), i.e., frame averages (15, 2, 1), are shown.

Frame averages		1	2	…	15
TV	$\lambda_{\text{adaptive}}$	166	74		14
	$\lambda_{\text{strong}}$	166	166		166
	$\lambda_{\text{medium}}$	42	42		42
	$\lambda_{\text{weak}}$	14	14		14
GAMMA	$\lambda_{\text{adaptive}}$	510	274		54
	$\lambda_{\text{strong}}$	510	510		510
	$\lambda_{\text{medium}}$	158	158		158
	$\lambda_{\text{weak}}$	54	54		54

### 2.6 Image Quality Evaluation

**Fig. 3:**
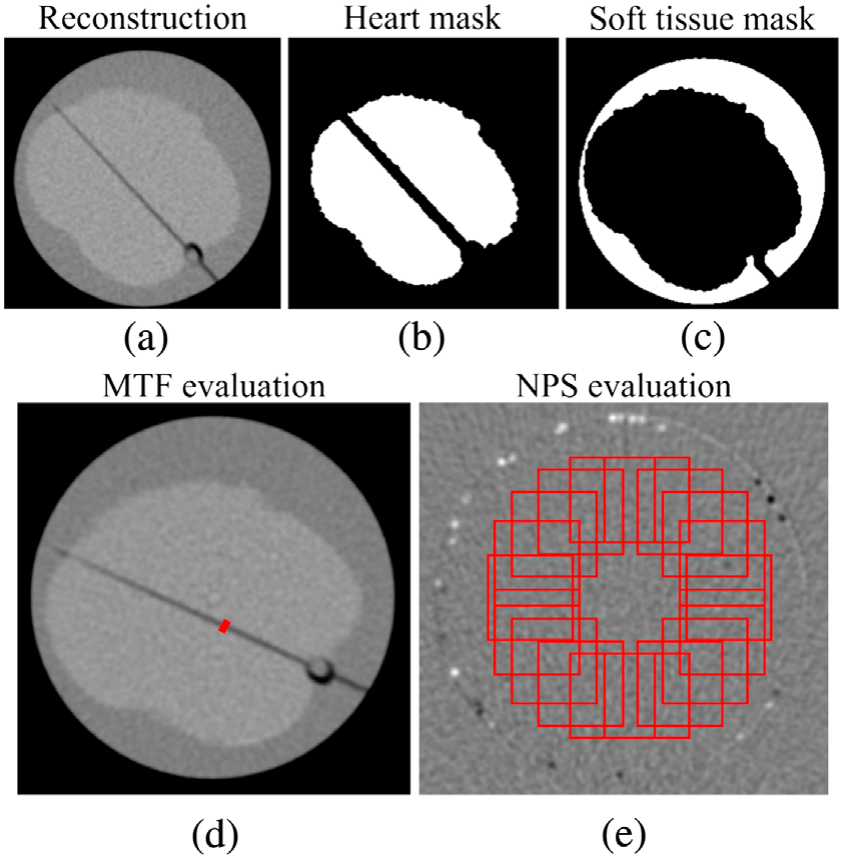
(a)–(c) Illustration of the region-of-interest (ROI) selection process for CNR evaluation. (d) MTF and (e) NPS ROI selection. NPS is evaluated from the subtraction image between neighboring slices.

To compare FBP and IR, we calculated the noise, determined as the standard deviation (SD) of HU values, and CNR between the heart and the soft-tissue background region of the cardiac phantom (Fig. 3).^50^ The heart and soft-tissue background regions-of-interest (ROIs) were segmented using Otsu’s method^51^ (Fig. 3). In addition to noise and CNR, we evaluated the radial NPS to compare noise texture between clinical CT, PC-FDCT, and between different IR methods and dose levels. The frequency range of the noise, characterized by the NPS, provides a comprehensive description of the noise characteristics.^52^ We measured the NPS by radially sampling ROIs (64×64  pixels) from the reconstructions of the homogeneous epoxy resin phantom [Figs. 1(b) and 3]. Three slices were used in NPS evaluation.

For the assessment of spatial resolution, MTF was estimated by Fourier transforming the line spread function originating from the air gap in the cardiac rod phantom (Fig. 3).^53^

### 2.7 Radiation Dose Measurements

We measured the air kerma of the PC-FDCT with a transparent detector (RaySafe Xi, Unfors RaySafe AB, Billdal, Sweden), which was positioned in a cylindrical 14-cm-diameter and 15-cm height PMMA phantom [Fig. 1(c)]. The air kerma was measured three times, and the mean and SD were reported. The air kerma values measured with this dose phantom were utilized to estimate the plausible air kerma levels encountered with these imaging settings and should not be considered as absolute values encountered in patient examinations or with using the 9-cm diameter imaging phantoms used in this study.

### 2.8 Statistical Analyses

Statistical analyses were performed with MATLAB. Hereafter, we refer to the different imaging protocols with the abbreviation FBP noise level/imaging system/reconstruction method. We used the CaHA volumes and Agatston scores from the FBP reconstructed LNP PC-FDCT scan (LNP/PCD/FBP) as ground truth values and compared them to the values obtained with FBP and IR at the reference FBP noise levels (CNP and HNP). The Wilcoxon signed-rank test (significance level p<0.05) was used for paired comparisons between reference LNP/PCD/FBP and higher noise protocols (HNP and CNP). The statistical significance of the CAC volume and score differences between the three different fixed IR regularization strengths was evaluated using the Friedman test (significance level p<0.05), and the Wilcoxon signed-rank test with Bonferroni correction was used for *post hoc* analysis (significance level p<0.017). Pearson correlation (r and p values) and Bland–Altman (BA) plots were used to compare the measured CAC scores and volumes between LNP, CNP, and HNP. Finally, the risk reclassification rate, i.e., the frequency of reassigned CAD grades for the calcifications, was used as a clinical indicator for the relevance of the CAC score differences.^31^ In the case of false positive, the true CAD grade of no CAD is reclassified to minimal, mild, moderate, or severe. Similarly, a false negative also results in reclassification. The CAD grades from LNP/PCD/FBP were used as ground truth grades.

## 3 Results

### 3.1 Characteristics of the Reference Noise Level Protocols

The air kerma for the low-noise (high-dose) PC-FDCT protocol was 1.475±0.003  mGy. Therefore, the 15 different dose levels that were obtained through different frame averages with PC-FDCT were 0.098, 0.197, 0.295, …, 1.475 mGy. The main properties of the reference noise protocols were derived based on these measured air kerma values (Table 4).

**Table 4:** Main characteristics for the reference noise level protocols.

Protocol	Number of frame averages	Noise (HU)^a^	Measured air kerma (mGy)^b^
LNP	15	7.45	1.475
CNP	2	18.95	0.197
HNP	1	27.45	0.098

a Measured from the cardiac rod phantom from FBP reconstruction.

b Determined from the cylindrical 14-cm diameter acrylic dose measurement phantom.

### 3.2 Quantification of CaHA

**Fig. 4:**
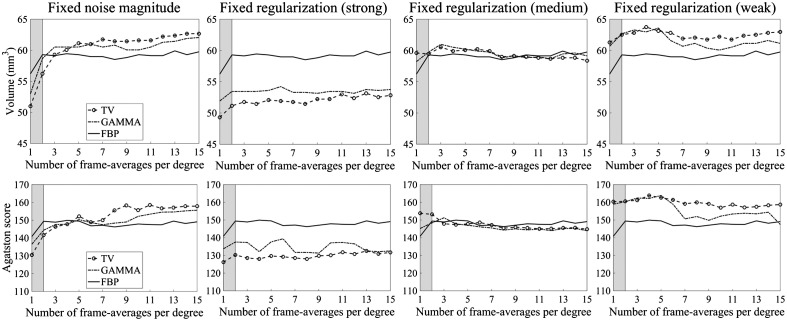
CAC volumes and scores with a different number of frame averages for $400\;\mathrm{mg}/{\mathrm{cm}}^{3}$, 3-mm-diameter calcification ($\text{true volume}=49.5\;{\mathrm{mm}}^{3}$). Shaded areas indicate the different noise ranges (gray: HNP–CNP and white: CNP–LNP).

**Fig. 5:**
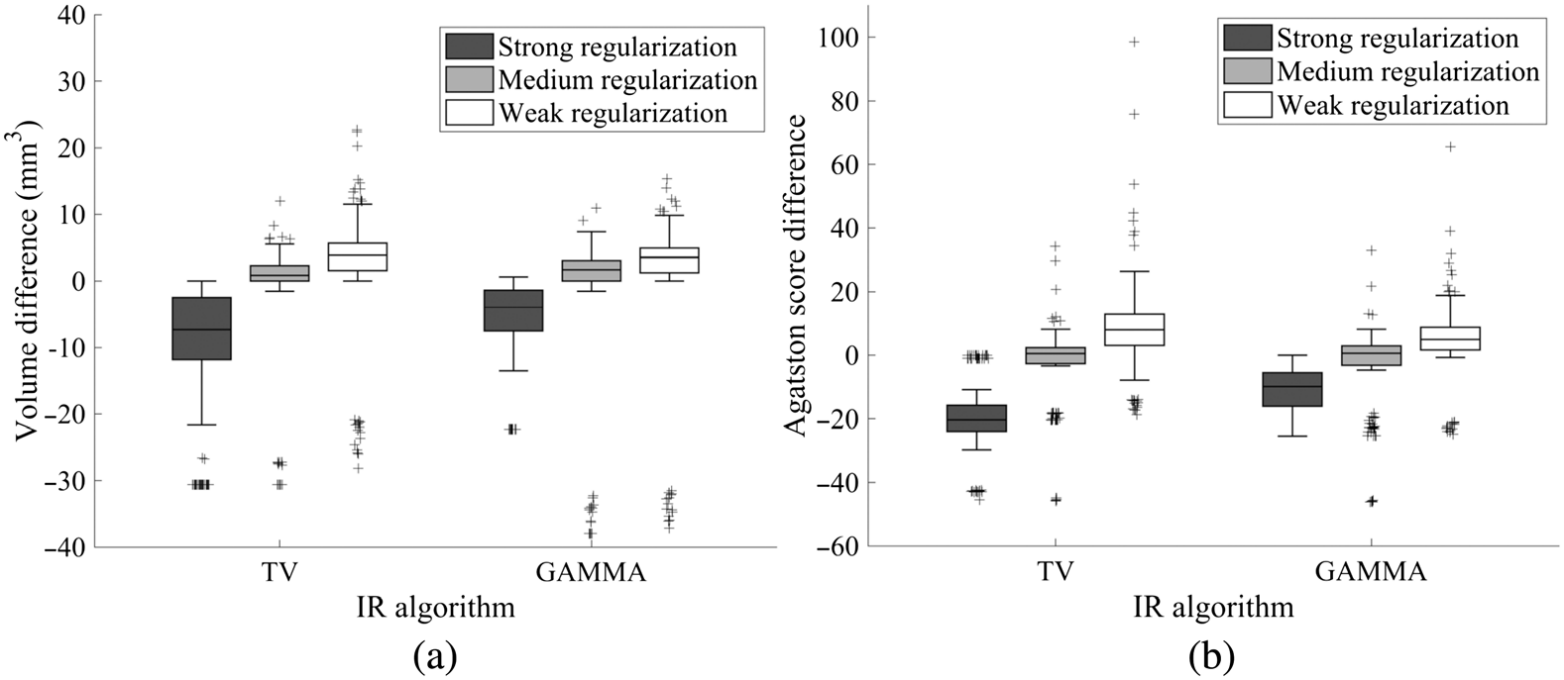
(a) CAC volume and (b) Agatston score differences between CNP iterative reconstructions and LNP FBP reconstruction.

Selection of the regularization parameter had profound effects on the CAC volumes and scores. First, using a fixed regularization parameter provided less variation in CAC volumes and scores with respect to dose compared to adaptive regularization parameter selection (Fig. 4). Second, the fixed regularization strength affected the CAC volumes and scores (Figs. 4 and 5) such that increasing the fixed regularization strength reduced the CAC volumes and scores significantly for both IR methods (p<0.05, Friedman). *Post hoc* analysis showed that the significant differences in scores and volumes were between strong and weak regularization strengths (p<0.017, Wilcoxon). Consequently, we chose the regularization parameter selection approach that produced the most comparable CAC volumes and scores with the reference LNP/PCD/FBP (fixed regularization parameter with medium regularization strength) and used those reconstructions for the remaining CAC quantification analyses.

The measured CAC volumes and scores for FBP with the reference dose levels are summarized in Table 5. The CAC scores ranged between 0 and 407 with the LNP. The measured CAC volumes were underestimated for low-density inserts, whereas the volumes of high-density inserts were overestimated (Table 5).

**Table 5:** Measured calcium volumes and Agatston scores for reference noise level PC-FDCT protocols with FBP.

Nominal density ($\mathrm{mg}/{\mathrm{cm}}^{3}$)	Insert diameter (mm)	Nominal volume (${\mathrm{mm}}^{3}$)	LNP			CNP			HNP		
			Volume (${\mathrm{mm}}^{3}$)	Score	Grade	Volume (${\mathrm{mm}}^{3}$)	Score	Grade	Volume (${\mathrm{mm}}^{3}$)	Score	Grade
400	5	137.4	156.6	407	Severe	158.5	409	Severe	155.6	404	Severe
400	3	49.5	59.8	149	Moderate	59.3	149	Moderate	56.2	141	Moderate
400	1.2	7.9	11.5	15	Mild	12.1	16	Mild	10.3	14	Mild
250	5	137.4	108.3	254	Moderate	108.3	253	Moderate	106.3	249	Moderate
250	3	49.5	39.6	77	Mild	38.5	75	Mild	36.1	64	Mild
250	1.2	7.9	3.2	2	Minimal	3.2	2	Minimal	0	0	No CAD
100	5	137.4	30.1	20	Mild	32.6	31.5	Mild	31.8	41	Mild
100	3	49.5	5.7	3	Minimal	3.5	2	Minimal	2.0	1	Minimal
100	1.2	7.9	0	0	No CAD	0	0	No CAD	0	0	No CAD

With CNP/PCD (0.20 mGy), significant differences were observed only in the scores of TV reconstructed calcifications when comparing to LNP/PCD/FBP (volume: $p_{\mathrm{FBP}}=0.69$, $p_{\mathrm{TV}}=0.07$, and $p_{\mathrm{GAMMA}}=0.22$ and Agatston score: $p_{\mathrm{FBP}}=0.78$, $p_{\mathrm{TV}}=0.03$, and $p_{\mathrm{GAMMA}}=0.10$, Wilcoxon). With HNP/PCD (0.098 mGy), on the other hand, statistically significant differences were observed both for each CAC volume and for the score with TV-reconstructed calcifications when comparing to LNP/PCD/FBP (volume: $p_{\mathrm{FBP}}=0.04$, $p_{\mathrm{TV}}=0.04$, and $p_{\mathrm{GAMMA}}=0.04$ and Agatston score: $p_{\mathrm{FBP}}=0.18$, $p_{\mathrm{TV}}=0.01$, and $p_{\mathrm{GAMMA}}=0.08$, Wilcoxon).

**Fig. 6:**
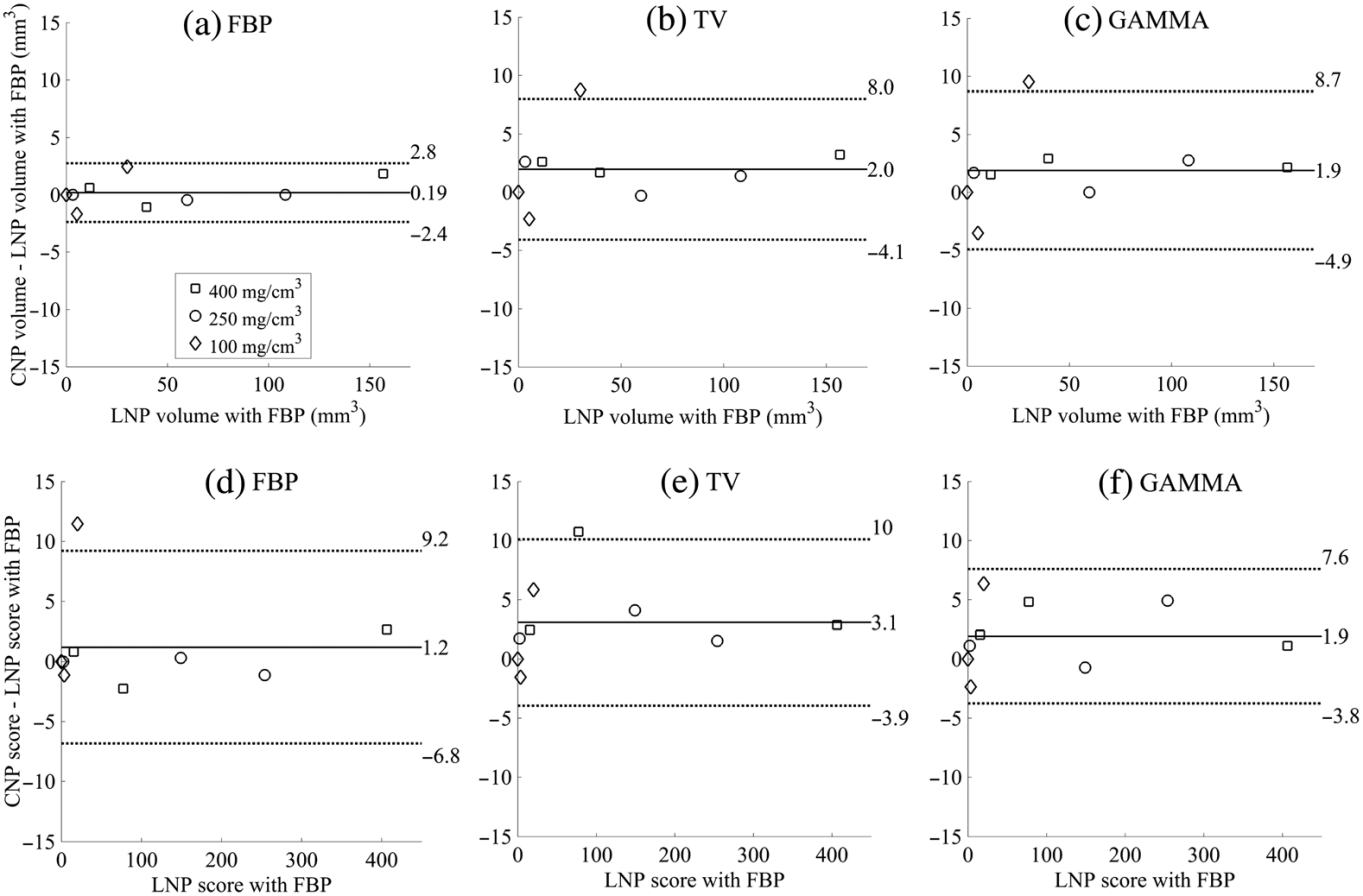
BA plots between ground truth LNP FBP and different reconstruction methods for the CNP for (a)–(c) CaHA volume and (d)–(f) Agatston score.

**Fig. 7:**
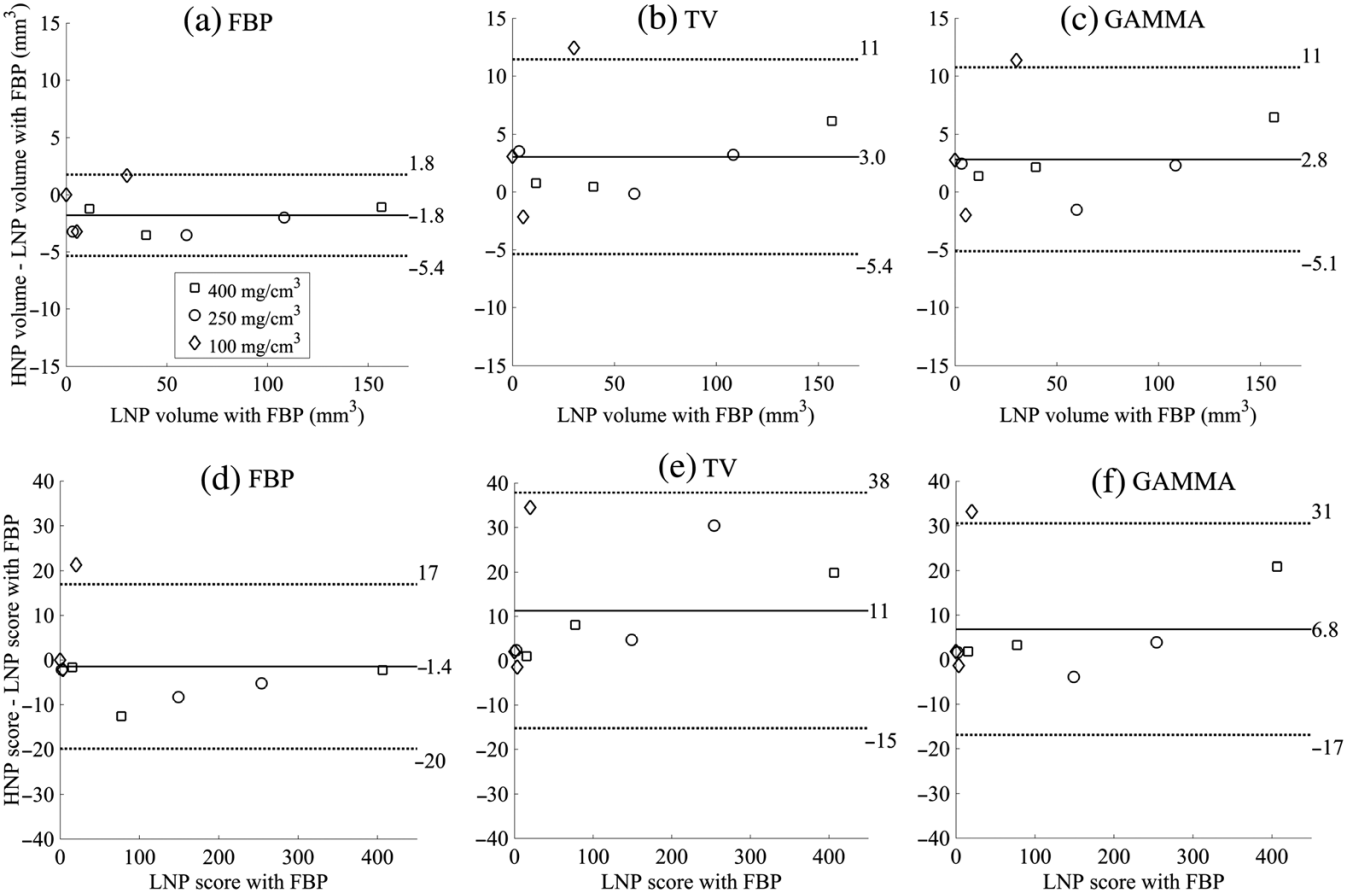
BA plots between ground truth LNP FBP and different reconstruction methods for the HNP for (a)–(c) CaHA volume and (d)–(f) Agatston score. Note the different y axis scales between CaHA volume and Agatston score.

FBP quantified the CAC volumes and scores with the highest accuracy, whereas IR had more evident differences as shown in the BA plots (Figs. 6 and 7). However, the CAC volumes and scores correlated strongly for each reconstruction method with the CNP/PCD when compared to the LNP/PCD/FBP volumes and scores (r=0.99 and p<0.05 for each method). For HNP/PCD, the CAC volumes and scores also correlated strongly with those of the LNP/PCD/FBP (r=0.99 and p<0.05 for each method).

CAD grade variation was the same for FBP and both IR methods, and their reclassification rates were 0% and 11.1% for CNP/PCD and HNP/PCD, respectively (Table 6). The reclassification rates over every dose setting were also highly comparable between the methods: FBP=3.2%, TV=7.9%, and GAMMA=4.8%.

**Table 6:** CAD grade reclassification rates for different FBP noise levels and reconstruction methods.

Dose level	Reclassification rate (%)		
	FBP	TV	GAMMA
CNP	0	0	0
HNP	11.1	11.1	11.1
Overall^a^	3.2	7.9	4.8

a Overall reclassification rate indicates the frequency of reclassification over every noise level.

### 3.3 Image Quality with IR

**Fig. 8:**
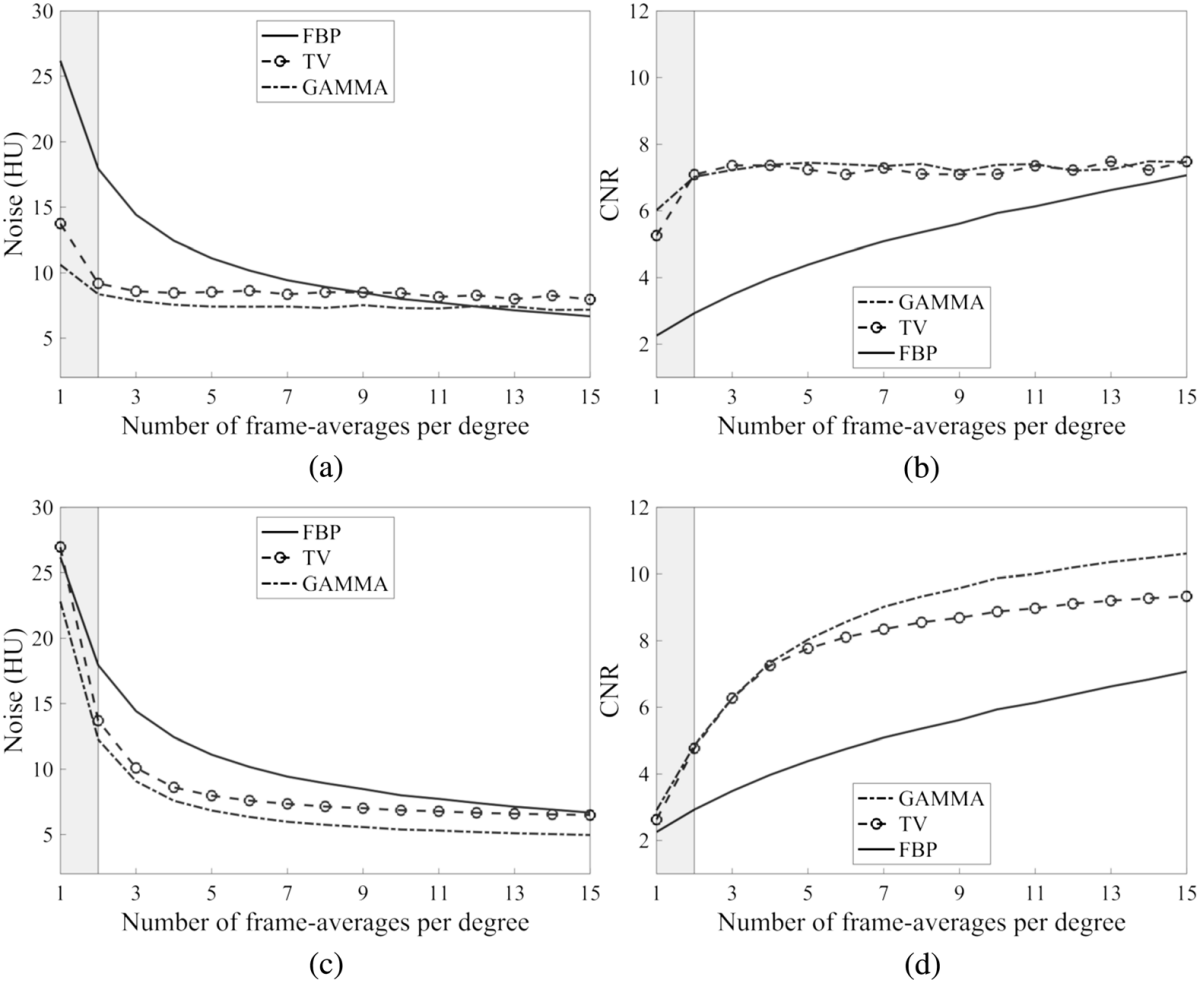
Measured noises and CNRs for PC-FDCT at a different number of frame averages. (a), (b) Image noise and CNR for adaptive regularization parameter selection. (c), (d) Noise and CNR for fixed regularization parameters (medium regularization strength). Shaded areas indicate the different FBP noise ranges (gray: HNP–CNP and white: CNP–LNP).

We observed higher CNR with IR over FBP for both fixed and adaptive regularization parameter selection approaches (Fig. 8). The CNR as a function of frame averages was similar between IR with fixed regularization parameters and FBP: CNR increased with an increasing number of frame averages (increasing dose) for each IR method [Figs. 8(c) and 8(d)]. With adaptive regularization parameter selection, on the other hand, CNR and noise magnitude were nearly constant throughout the used dose range [Figs. 8(a) and (b)].

**Fig. 9:**
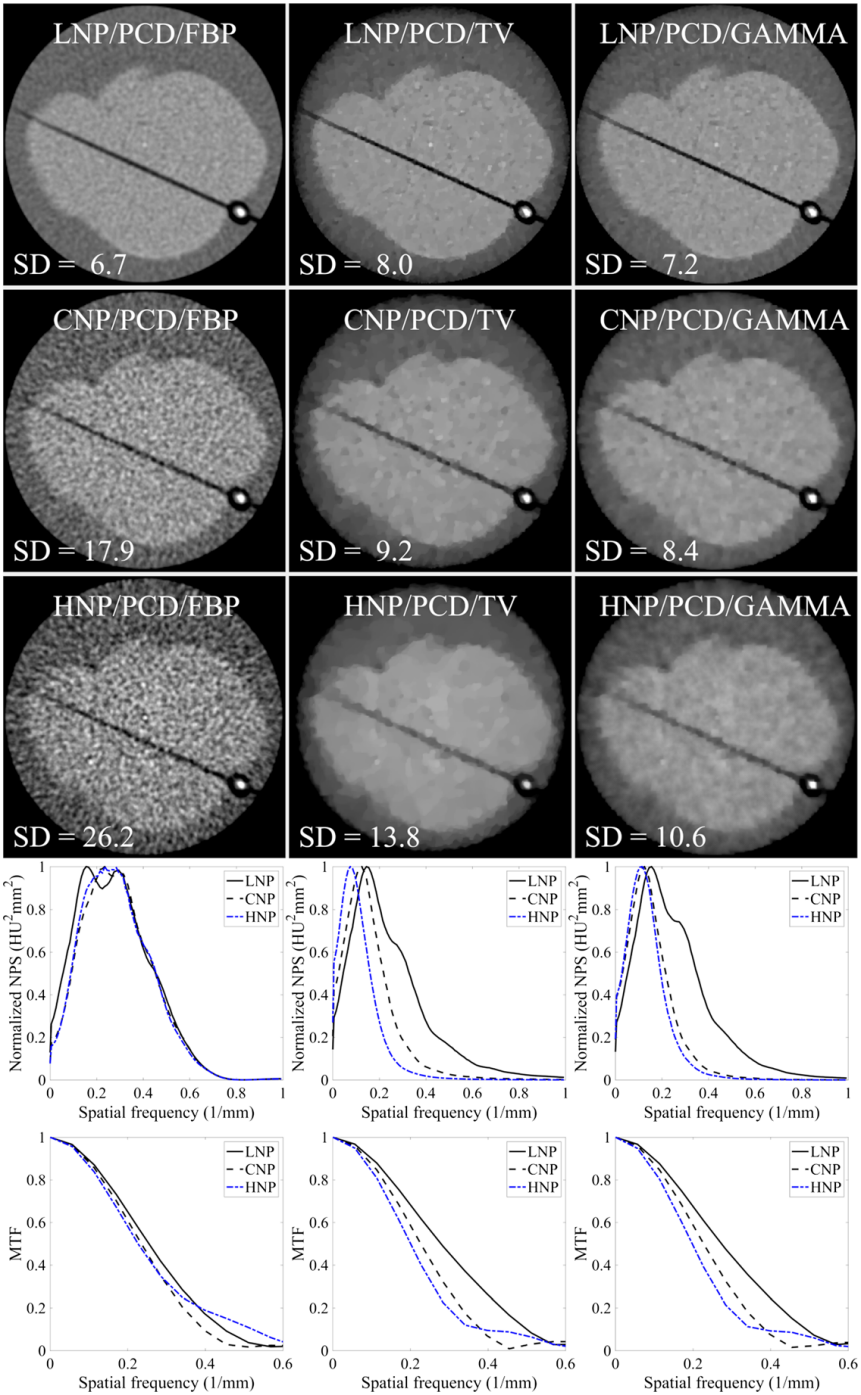
Illustrations of reconstructions with LNP, CNP, and HNP, and their respective normalized NPSs, noise SDs, and MTF curves. IRs with adaptively selected regularization parameters are visualized. CaHA insert with $400\text{-}\mathrm{mg}/{\mathrm{cm}}^{3}$ density and 1.2-mm-diameter is shown in the reconstructions. Windowing for reconstructions was [−100,150]  HU. Noise SDs between the LNP reconstructions differ since the noise was matched to that of the epoxy resin phantom. The noise power spectra deviate substantially between IR and FBP.

IR provided improvements in technical image quality, as illustrated by the increased CNR values, improved MTF, and visually sharper images, when compared to LNP/PCD/FBP and CNP/PCD/FBP (Figs. 8 and 9). However, the NPSs and MTFs deviated substantially between FBP and the different IR methods, producing visually apparent differences in the observed noise-texture and image sharpness between reconstructions (Fig. 9).

**Fig. 10:**
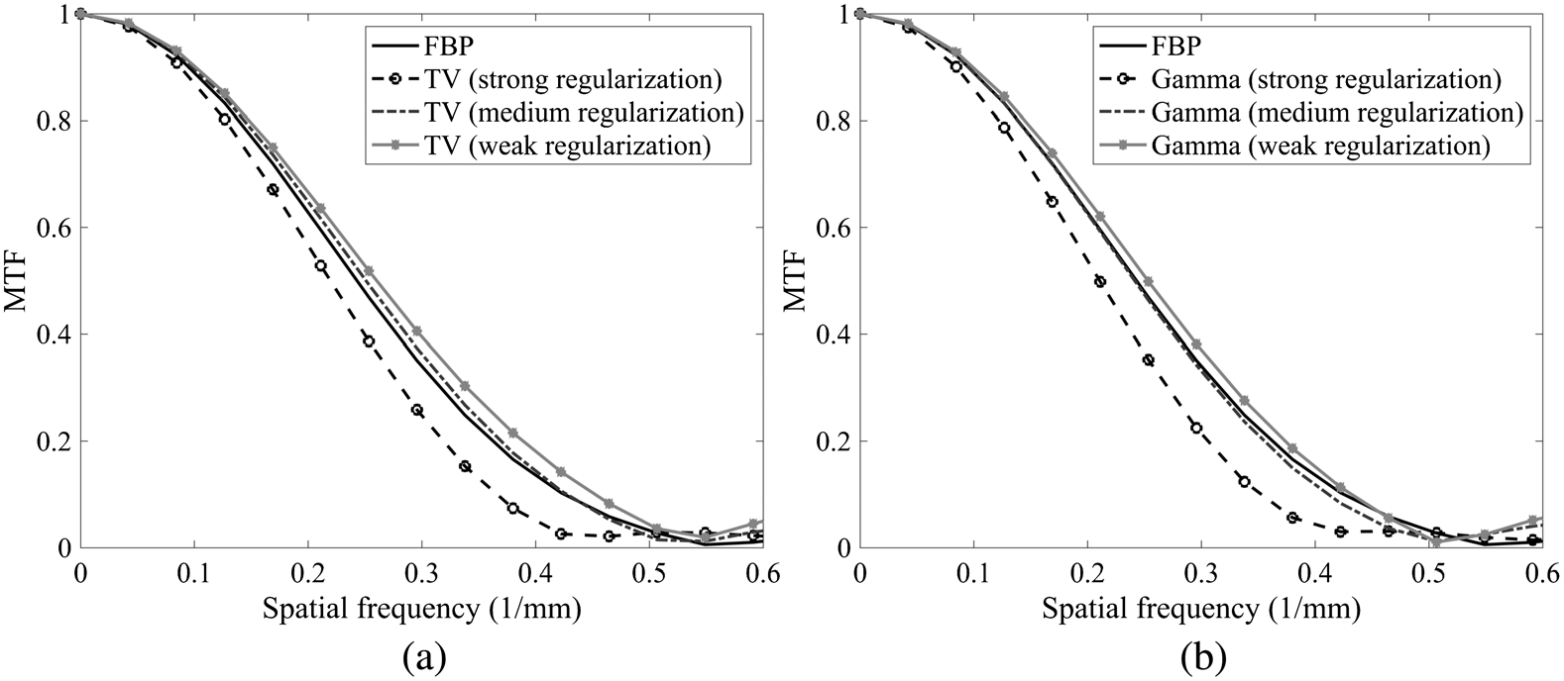
MTFs for LNP reconstructions with different fixed IR regularization strengths: (a) TV regularization and (b) Gamma regularization.

As increasing IR regularization strength produced significantly different CAC volumes, CAC scores, and more blurred CaHA insert reconstructions, we decided to compare the MTFs between LNP/PCD/FBP and LNP/PCD/IR with different fixed regularization strengths (Fig. 10). Interestingly, the regularization strength that was chosen to yield the most similar MTF with FBP (medium regularization strength) also produced the most comparable CAC volumes and scores.

## 4 Discussion

The purpose of this study was, first, to investigate the preservation of PC-FDCT CAC scores of CaHA inserts with IR, and second, to evaluate whether CAC scoring can be performed with low radiation dose with IR. With a simplified model for CAC scoring protocol, we demonstrated that accurate CaHA volume quantification and CAC scoring with IR is feasible with the appropriate selection of regularization parameters and that the CAD grade reclassification rate with IR was comparable to that of the FBP. Moreover, the reclassification rates with IR at the reference FBP noise levels (CNP=0% and HNP=11.1%) and the overall reclassification rates over every noise level (TV=7.9% and GAMMA=4.8%) were within the clinical CAD grade variation caused by merely changing the starting position of a scan (10% to 11%).^32^^,^^33^

Regarding low-dose CAC scoring, no dose-reduction possibility was observed with IR, and FBP demonstrated improved preservation of CAC scores with the HNP. Nonetheless, dose reductions may still be achievable with model-based IR^30^ or other approaches. For example, deep learning reconstruction has been shown to improve the image quality compared to the methods used in this study.^47^

With the CNP, IR produced slightly more variation in the CAC volumes but yielded lower SD in the Agatston scores compared to FBP. Iterative noise reduction with IR may produce reconstructions, whose volume estimates are more susceptible to partial volume effects compared to the Agatston score. This may explain the observation that despite the slightly larger CAC volume deviation in BA plots (Fig. 6), the CAC scores had a smaller SD with IR compared to FBP. Similar outcomes have also been reported in prior studies, in which coronary mass scores were better preserved than CAC volumes with IR.^27^^,^^28^ Finally, we underlined the importance of MTF in the CAC scoring accuracy of IR algorithms.

Consistently with existing literature, increasing regularization strength was observed to significantly reduce both CAC volumes and Agatston scores.^26^^,^^27^ We identified deviating spatial resolution (MTF) as the key factor for this phenomenon, the CAC volume and CAC scoring accuracy were optimal with an IR regularization strength that produced most comparable MTF with LNP/PCD/FBP. The discrepancy in the CAC scoring performance of IR in prior research may be due to mismatched MTFs between FBP and IR. The deviating image sharpness is naturally not the sole factor influencing CAC scoring performance since, e.g., image noise can affect the CAC score.^26^ In this study, however, the increased noise present in the HNP/PCD and CNP/PCD images did not substantially alter the CAC volume and score estimates with either FBP or IR with fixed regularization strength. This observation indicates that the noise is not as substantial a factor in CAC scoring accuracy as the blurring of the CAC boundary caused by the increased regularization strength. Consequently, the regularization strength should be fixed with CAC scoring IR, and it should not be adapted, e.g., based on patient size, to reduce image noise with larger patients since changing the regularization strength would deteriorate the CAC scoring accuracy, as shown in this study.

The fixed threshold of 130 HU in CAC scoring is known to affect the volume scores by overestimating the volumes of very dense calcifications and underestimating the volumes of less dense calcifications.^54^ We observed similar density dependence in volume-estimation in this study: measured volumes were larger for high-density inserts compared to low-density inserts with the same nominal size.

IR improved the image quality in terms of CNR and MTF when compared to FBP with the same radiation dose. However, the NPSs (noise textures) were different between IR and FBP, making a direct image quality comparison between the methods extremely difficult. As CNR, NPS, and MTF all influence the image quality, we decided to report these parameters for the reference FBP noise levels used in the literature. Furthermore, regularization parameter selection also plays a vital role in the observed image quality of IR. We chose the parameters that (1) result in a fixed noise magnitude and (2) are fixed to one value for each frame average setting. As an example, fixing the noise magnitude resulted in nearly constant CNR with respect to the number of frame averages, whereas the reconstruction obtained using a fixed regularization parameter resulted in more comparable CNR to FBP. Using CNR as the sole image quality measure can, therefore, be deceptive as adaptive regularization preserves the CNR even at extremely low radiation doses, although the visual image quality is deteriorating. This approach prefers over-regularized images with a more “paint-brushed” appearance, which was also observed in the reconstructions and NPSs of the HNP.

We observed considerable variation in the noise magnitudes of the reference clinical protocols between different studies. In our literature review, the noise range of the reference clinical protocols was between 14 to 20.7 HU, so the number averaged frames per degree for the CNP/PCD protocol in our study could have been between 2 (SD=19.0  HU) and 4 (SD=13.4  HU).^29^^,^^30^^,^^55^ We decided to follow the guideline of 20 HU target noise level for CAC scoring.^43^ However, for PC-FDCT, with potentially larger scanned field-of-views, and consequently increased scattering, this target noise level may be suboptimal.

Some limitations exist in this study. We used a cardiac rod phantom instead of a torso to avoid the interior tomography problem that would arise since the torso would not fit into the small active area of the PCD. This cylinder phantom attenuates less than the human torso, and the radiation exposures used in this study would result in substantially worse image qualities with a torso due to increased attenuation, beam-hardening, and x-ray scattering. We mitigated this issue by fixing the PC-FDCT reference noise levels to values that produced similar noise magnitudes as have been reported in the previous studies. Other limitations are the lack of cardiac motion and the deviating imaging geometry and equipment between the clinical CT scanner and our PC-FDCT setup. Owing to the high dead time with PCDs,^56^ the x-ray flux may have to be limited with PCD-CT, resulting in longer scans and increased cardiac motion, which may impair the image quality. Furthermore, the deviating imaging geometry between the PC-FDCT and EID-CT has to be addressed. The lack of existing PCDs with detector arrays in a curved geometry limited the analysis to a flat-panel detector, which may influence the reconstruction and image quality due to plausible cone-beam artifacts. However, in this study, we limited the beam magnification to reduce this artifact and treated the imaging geometry as a fan-beam geometry. Unfortunately, we could not directly install a dedicated curved PCD into a clinical CT scanner, so we could only mimic the imaging parameters, such as beam collimation, of clinical CT. Consequently, we presented direct dose-and image-quality assessment between PC-FDCT and CT as an appendix since it has already been conducted with more comparable imaging setups.^7^^,^^10^^–^^15^ Finally, owing to the deviating diameters between the imaging phantoms and the phantom used for determining the air kerma, the measured air kerma values could only be utilized to estimate the true air kerma with the imaging phantoms.

The small number of calcification inserts (N=9) was also a notable limitation in this study. Nonetheless, we believe that the importance of IR regularization strength on the CAC scoring performance was demonstrated even with this small number of calcifications, which was representative of the range of different CAD grades. Finally, we only applied image quality parameters obtained from physical measurements. Other possible measures for image quality, such as visual grading analysis by a radiologist or model observers,^53^ could be diagnostically more descriptive measures than the physical parameters (CNR, noise magnitude, NPS, and MTF) used in this study.

Future research should investigate the possible dose reductions when using a torso phantom and interior tomography imaging geometry.^57^ This geometry can induce further dose reductions,^5^ but it requires additional image processing, such as sinogram extension techniques,^58^ to reduce the characteristic truncation artifacts originating from the limited field-of-view. Further research is also needed to expand these results for more realistic cardiac PC-FDCT and clinical CT studies with the torso and a moving heart rather than a cylindrical cardiac rod phantom. Additionally, since PCDs influence the image contrast, optimizing the Agatston scoring threshold value for PCD-CT is an interesting topic for further investigations. Furthermore, the spectral information from PCD could be exploited to quantify the CaHA volume, density, and mass more accurately.^37^ Validating the observed relation between MTF and CAC quantification accuracy between IR and FBP is also an important task to perform with clinical scanners and clinical IR algorithms.

## 5 Conclusions

IR regularization strength is an important determinant of CAC scoring performance. We showed that CAC quantification of CaHA inserts between FBP and IR could yield comparable outcomes when IR regularization strength was optimized to produce a similar spatial resolution (MTF) to reference FBP reconstruction. On the other hand, the image noise was not a critical factor in the CAC scoring accuracy of IR. Therefore, we suggest that the regularization strength of IR for CAC scoring applications should be tuned such that the MTFs between IR and the CAC scoring FBP kernel match.

However, no improvement at low-dose CAC scoring was observed with IR, and FBP produced the most accurate CAC volumes and scores at the lowest dose setting. Consequently, this study did not demonstrate improved CAC scoring accuracy at a low dose with the utilized IR methods.

## 6 Appendix A

**Fig. 11:**
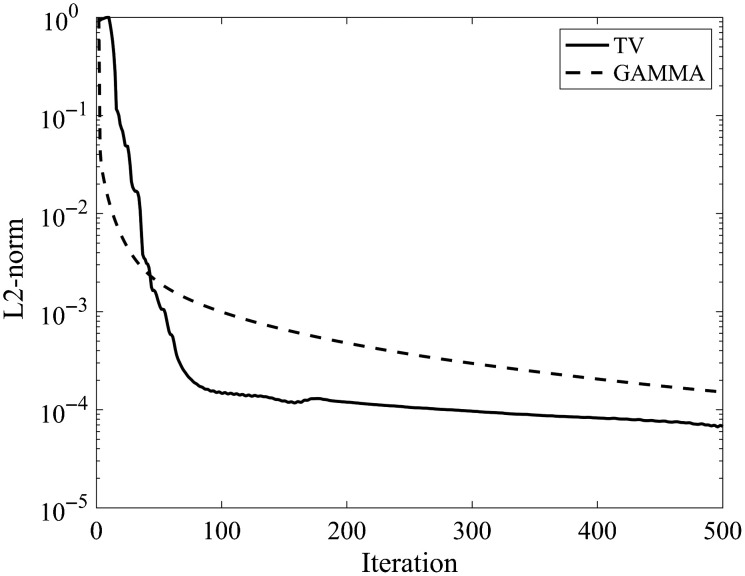
Convergence for TV minimization and gamma regularization (GAMMA). The vertical axis is in logarithmic scale. $L_{2}$-norm was calculated between reconstructions of consecutive iterations.

We monitored the $L_{2}$-norm between reconstructions of consecutive iterations to verify that the reconstruction had reached an accurate solution (Fig. 11).

## 7 Appendix B

This section provides a comparison of image quality, CaHA quantification accuracy, and radiation dose between a clinical CT scanner and our experimental PC-FDCT setup. We measured the cardiac rod phantom with both CT and PC-FDCT to make a fair comparison of image quality and dose. We used a clinical CT scanner (Somatom Definition Flash, Siemens Healthcare, Erlangen, Germany) in axial mode with a coronary calcium scoring protocol using the lowest exposure setting (10 mAs). We used this lowest exposure setting since it provided the most comparable noise magnitude (SD=8.2  HU) with the CAC scoring guideline value of 20 HU. The other main imaging parameters of this protocol are summarized in Table 1.

**Fig. 12:**
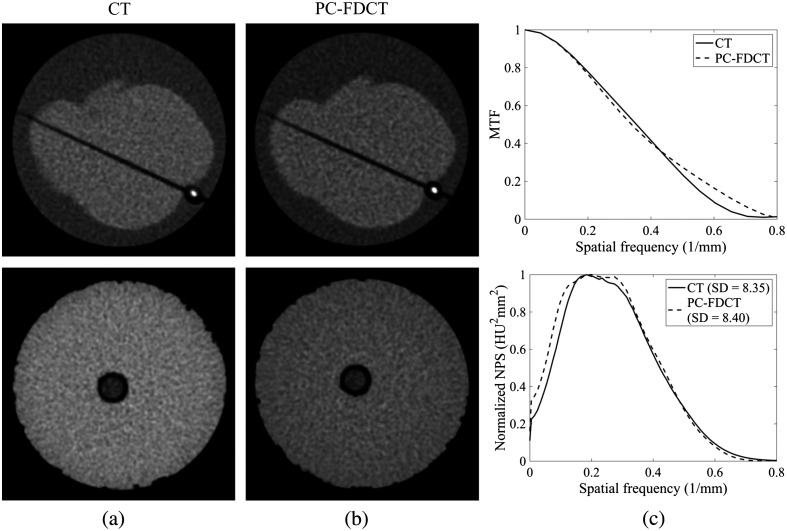
CT and PC-FDCT reconstructions of the cardiac rod and epoxy resin phantoms with similar image quality (image noise) using FBP and their corresponding normalized NPSs with noise SD (estimated from the epoxy resin phantom). The MTFs were estimated from the cardiac rod phantom. (a) Clinical CT reconstructions (air kerma of 1.090 mGy); (b) PC-FDCT reconstructions with air kerma of 0.885 mGy; and (c) MTF and normalized NPS. Calcification with a density of $400\;\mathrm{mg}/{\mathrm{cm}}^{3}$ and a 3.0-mm diameter is visualized. Windowing was set at [−100,150]  HU.

The image noise and visual appearance between clinical CT and PC-FDCT reconstructions of the cardiac rod and epoxy resin phantoms were matched with nine-frame averaged PC-FDCT reconstruction (Fig. 12). The corresponding air kerma values were 1.090 and 0.885 mGy for CT and PC-FDCT, resulting in an 18.8% dose reduction with PC-FDCT. Overall, the CT and PC-FDCT reconstructions appeared visually similar, disregarding a slight difference at low spatial frequencies due to a small ringing artifact with PC-FDCT (Fig. 12). The MTF was slightly improved with PC-FDCT.

With similar air kerma values for CT (1.090 mGy) and PC-FDCT (1.082 mGy and 11 frame averages), the image noises were ${\mathrm{SD}}_{\mathrm{CT}}=7.91\;\mathrm{HU}$ and ${\mathrm{SD}}_{\mathrm{PC}\text{-}\mathrm{FDCT}}=7.01\;\mathrm{HU}$ for the cardiac rod phantom, corresponding to a decrease of 11.4% in image noise with PC-FDCT.

In CaHA quantification, a clear deviation, characterized by a substantial volume underestimation with decreasing calcification density, was observed for both CT and PC-FDCT between nominal and measured calcium volumes (Table 7). Larger calcification volumes and higher Agatston scores were measured with PC-FDCT compared to CT.

**Table 7:** Measured calcium volumes and Agatston scores for CT and PC-FDCT (nine-frame averages per degree) with FBP.

Nominal density ($\mathrm{mg}/{\mathrm{cm}}^{3}$)	Insert diameter (mm)	Nominal volume (${\mathrm{mm}}^{3}$)	CT		PC-FDCT	
			Volume (${\mathrm{mm}}^{3}$)	Score	Volume (${\mathrm{mm}}^{3}$)	Score
400	5	137.4	146.1	363	152.4	396
400	3	49.5	47.8	113	57.2	144
400	1.2	7.9	6.0	4	10.6	14
250	5	137.4	87.1	158	104.6	223
250	3	49.5	46.1	112	38.2	75
250	1.2	7.9	0	0	0	0
100	5	137.4	22.6	15	24.4	16
100	3	49.5	1.5	1	1.8	1
100	1.2	7.9	0	0	0	0

The measured noise-reduction with PC-FDCT is comparable to previous research with PCD-CT, in which noise reductions from 5% to 20% have been reported when compared to conventional CT with the same radiation dose.^7^^,^^10^^–^^15^

In conclusion, PC-FDCT provided comparable image quality to EID-CT with a reduced dose.
